# Iron-sulfur flavoenzymes: the added value of making the most ancient redox cofactors and the versatile flavins work together

**DOI:** 10.1098/rsob.210010

**Published:** 2021-05-05

**Authors:** Maria Antonietta Vanoni

**Affiliations:** Dipartimento di Bioscienze, Università degli Studi di Milano, Via Celoria 26, 20133 Milano, Italy

**Keywords:** flavin coenzymes, iron-sulfur clusters, oxido-reduction, electron transport, enzymes

## Abstract

Iron-sulfur (Fe-S) flavoproteins form a broad and growing class of complex, multi-domain and often multi-subunit proteins coupling the most ancient cofactors (the Fe-S clusters) and the most versatile coenzymes (the flavin coenzymes, FMN and FAD). These enzymes catalyse oxidoreduction reactions usually acting as switches between donors of electron pairs and acceptors of single electrons, and vice versa. Through selected examples, the enzymes' structure−function relationships with respect to rate and directionality of the electron transfer steps, the role of the apoprotein and its dynamics in modulating the electron transfer process will be discussed.

## Graphical abstract

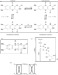

## 1. Introduction

Flavoenzymes form a broad and growing class of proteins with diverse, and even unexpected, biological roles including primary energy metabolism, synthesis of key cell constituents and secondary metabolites, xenobiotic degradation/detoxification, synthesis and degradation of neurotransmitters and coenzymes, DNA repair, gene expression regulation, control of the circadian clock and magnetoreception, generation of (and protection from) reactive oxygen and nitrogen species, control of cell contacts, migration, differentiation and duplication, to name a few. For recent reviews, see [1–5] and references therein.

**Figure 1.**
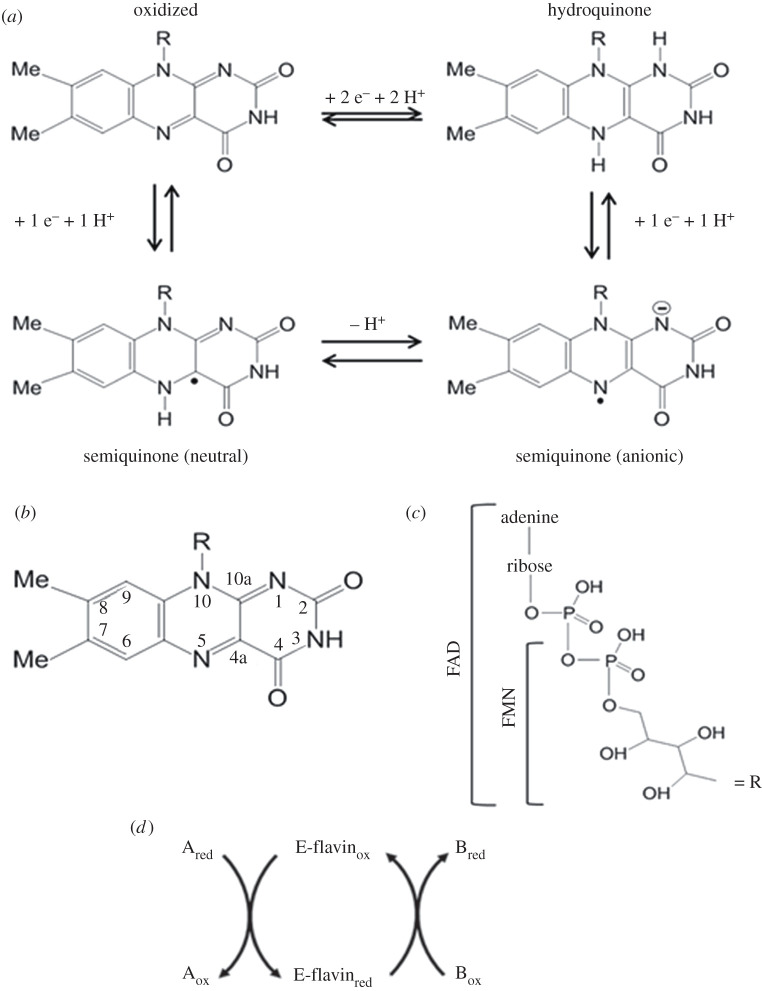
The Ox, Sq and Hq forms of flavin coenzymes. (*a*) Structures of the Ox, Sq and Hq forms of flavin coenzymes. In the Ox species, the N(3)H position may deprotonate depending on the protein environment; in the 2-electron reduced Hq form N(1)H may deprotonate; each one of the Sq forms is depicted reflecting one of the possible tautomers, and it is the protein environment that modulates the stabilization of the Sq as well as the electronic distribution. (*b*) Numbering of the isoalloxazine ring. (*c*) The N(10) substituent in FMN and FAD. (*d*) General mechanism of flavoenzyme-catalysed reaction formed by an enzyme reductive half reaction and an oxidative half reaction.

The success of the most common flavin coenzymes, flavin mononucleotide (FMN) and flavin adenine dinucleotide (FAD; figure 1), which are all derivatives of vitamin B_2_, as prosthetic groups is believed to mainly reside in their remarkable versatility with respect to the reactive positions of their isoalloxazine ring, the allowed redox states and the sensitivity of their reactivity, and corresponding oxidoreduction potentials, to the microenvironment. Their primary function is to participate in oxidoreduction (redox) reactions, but flavoenzymes with no net flavin oxidoreduction cycle have been found [1,4,6–8].

The flavin coenzymes exist in the oxidized (Ox), 1-electron reduced (semiquinone, Sq) and 2-electron reduced (hydroquinone, Hq) forms (figure 1). By being able to donate/accept electron pairs (i.e. hydride anions) or single electrons, they can act as switches between obligate donor/acceptors of single electrons or electron pairs. Furthermore, the ability to stabilize the 1-electron reduced species allows flavin-dependent enzymes to reduce molecular oxygen in oxidase, monooxygenase and dioxygenase reactions. How the protein controls the oxygen reactivity of flavin coenzymes is the focus of several lines of research (e.g. [9] for a review). The unpaired electron in 1-electron reduced flavins is a key feature that allows flavoproteins to also function as photoreceptors or even magnetoreceptors in biological phenomena such as circadian cycle control or bird migration, respectively [10,11].

In all cases, the redox potential of the flavin oxidized/2-electron reduced (Ox/Hq), oxidized/1-electron reduced (Ox/Sq) and 1-electron/2-electron reduced (Sq/Hq) couples and, therefore, the stability of each one of these species is finely tuned by the microenvironment of the flavin coenzyme itself. Thus, the stabilities of the Ox, Sq and Hq forms of the flavin depend on a variety of factors, such as solvation of the active site and protonation state of active site residues, overall electrostatics through long-range interactions, the presence of substrates/products in the active site and (even subtle) conformational changes induced by bound substrates or allosteric modulators, or which are integral part of the catalytic cycle.

The versatility of the flavin coenzymes and the variety of reactions they catalyse also make flavoenzymes of great interest for biotechnological and biomedical applications, such as in the context of biological synthesis of relevant compounds and as reagents in diagnostics and drug targets, respectively.

Flavoenzymes typically catalyse the overall reaction via two half reactions, which can be studied separately. In the enzyme reductive half reaction, one of the substrates is oxidized and the enzyme-bound flavin is reduced; in the oxidative half reaction, the coenzyme is reoxidized upon electron transfer to the second substrate (figure 1). Flavoenzymes have been initially classified on the basis of the overall catalysed reaction as: (i) transhydrogenases, which transfer 2-electron equivalents along with hydrogen ions from one organic substrate to another; (ii) dehydrogenase-oxidases, which accept 2-electron equivalents from an organic substrate, and molecular oxygen is reduced to hydrogen peroxide in the enzyme oxidative half reaction; (iii) dehydrogenase-monooxygenases, which are reduced by an organic substrate, typically a pyridine nucleotide (NADH or NADPH), and then reduce molecular oxygen leading to incorporation of one oxygen atom in the second substrate and of the second in a water molecule; (iv) dehydrogenase-electron transferases, which are reduced by 2-electron transfer from an organic substrate, and, then, are reoxidized by sequential 1-electron transfer steps to acceptors such as cytochromes or iron-sulfur (Fe-S) proteins; and (v) electron transferases, which are reduced and oxidized in 1-electron transfer steps [12,13]. However, several complex flavoproteins escape such classification belonging at the same time to at least two functional classes, as the result of the assembly of multiple domains, with the reaction taking place at distinct catalytic subsites. Therefore, an attempt to classify flavoenzymes, focusing on the types of enzyme oxidative and reductive half reactions that are mixed and matched to carry out various overall reactions, has been recently proposed in the extensive survey of Fagan & Palfey [1]. A relation between protein fold and catalysed reaction is often found most likely as the result of divergent evolution from common ancestors, but with exceptions. As an example, the recently discovered MICAL1 protein, although structurally closely related to the bacterial p-hydroxybenzoate hydroxylase, the prototype of NADH-dependent aromatic monooxygenases, appears to be a bona fide NADPH oxidase. However, it may exploit the remarkable conformational flexibility of p-hydroxybenzoate hydroxylase to provide an extra handle for activity regulation, and to adapt to the actin filament in its unprecedented actin-depolymerizing activity [14].

Several flavoenzymes use one or more additional redox centres to carry out their cognate reaction, like a second flavin coenzyme, haem, pterin, metal centres, and, often, one or more Fe-S clusters.

Early examples include succinate dehydrogenase (SDH, respiratory complex II) and the related fumarate reductase (FumR), xanthine oxidoreductase (XOR) and dihydroorotate dehydrogenase (DHODH) [15].

Why obligate 1-electron acceptors have been recruited in addition to the flavin coenzyme to carry out reactions when the flavin would, alone, be sufficient, being able to switch between mono- and bi-electronic transfer steps, is a frequent question in the field. Most likely, the complexity of some multi-redox centre enzymes results from random gene fusion/rearrangement events. Evolution through the selection of the ‘successful’ mutations eventually led to molecular machines capable to carry out a given reaction with sufficient efficiency to sustain life and, at the same time, to give room for different layers of control of the reaction in the cell (e.g. conformational changes occurring during the catalytic cycle that control redox potential and distances among redox centres), and also to prevent harmful reactions like those of reduced species with molecular oxygen (e.g. [16,17]). Indeed, it appears that no systematic studies of the evolutionary history of these proteins are available yet, although attempts have been made in some instances [18–20].

In the light of a large number of known Fe-S flavoenzymes, in the following, after an initial overview of the general properties of the prosthetic groups, selected examples of Fe-S flavoenzymes will be described to highlight different solutions found in nature to carry out and control certain reactions while exploiting the versatility of the flavin coenzymes. In particular, how efficient electron transfer across a broad range of donor/acceptor couples can be achieved by exploiting the ability of flavoenzymes to finely tune the stability of the Sq intermediate will be discussed. Indeed, the role of Sq stabilization to allow for electron transfer at a low potential from higher potential reductants such as NAD(P)H has recently come back into the limelight when flavin-based electron bifurcation (FBEB) has been demonstrated in a series of enzymes as a recently discovered mechanism of energy conservation [21,22].

## 2. The flavin coenzymes

The common flavin coenzymes (FAD and FMN; figure 1) derive from riboflavin (Rf, vitamin B_2_) by the action of riboflavin kinase (RK) leading to FMN, which is converted to FAD by adenyl transfer from ATP catalysed by FAD synthetase (FADS). RK and FADS can be independent units or a RK-FADS bifunctional enzyme may be found [23]. Trafficking and recycling of Rf, FMN and FAD is complex and, in humans, defects in any one of the players may lead to important disease [24,25].

The isoalloxazine ring of the flavin is the reactive part of the coenzyme, although its redox properties and reactivity are modulated also by interactions with other parts of the molecule, such as the 2′OH function of the ribityl side chain, besides protein side chains allowing for the variety of reactions involving flavin coenzymes [5,8,26–28].

**Figure 2.**
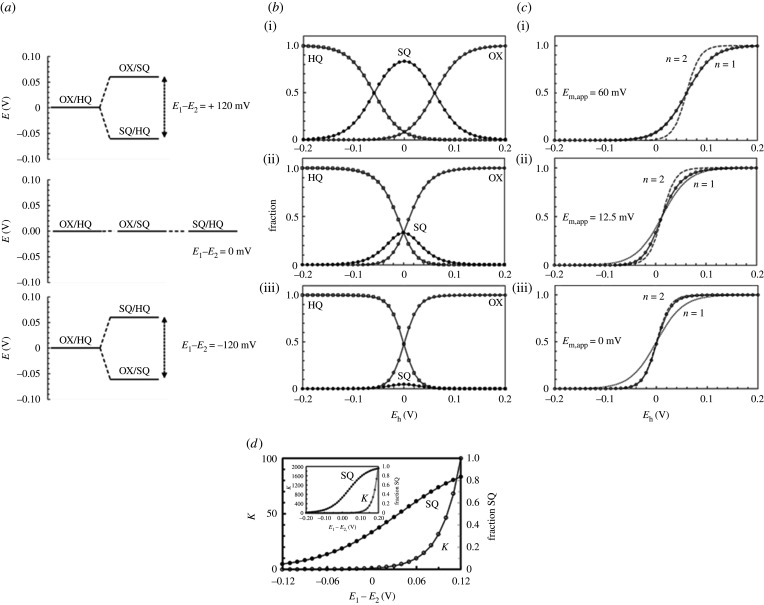
Effect of the potential difference between the Flavin_Ox_/Flavin_Sq_ (*E*_1_) and Flavin _Sq_/Flavin_Hq_ (*E*_2_) couples on the distribution of Ox, Sq and Hq forms of flavin coenzymes as a function of the potential. The figure shows the fraction of the oxidized, 1-electron reduced (Sq) and 2-electron fully reduced (Hq) flavin coenzyme as a function of the potential (*E*_h_) when the difference between the redox potential values of the Flavin_ox_/Flavin_sq_ (*E*_1_) and of the Flavin_sq_/Flavin_hq_ (*E*_2_) couples is +120 mV (top row), 0 mV (middle row) and −120 mV (bottom row). All redox potentials are calculated at pH 7 against the standard hydrogen electrode under standard conditions. The temperature has been set at approximately 30°C where the 2.203RT/nF term of the Nernst equation for 1 electron transfer is 0.06. For simplicity, the standard redox potential of each couple (*E*°′) has been indicated as *E*, and that of the Flavin_Ox_/Flavin_Hq_ couple, which results from ½ (*E*_1_ + *E*_2_) has been set to 0 mV. (*a*) Depicts the three cases taken into account. (*b*) Reports the fractional concentration of the Ox, Sq and reduced flavins in solution as a function of E_h_. (*c*) Shows how, in some cases, information on the presence of a Sq intermediate may be inferred from reductive titrations, even when—for any reason—only the Ox form can be monitored. When $E_{\mathrm{Ox}/\mathrm{Sq}}^{{{\;}^{\circ }}^{\prime }}\gg E_{\mathrm{Sq}/\mathrm{Hq}}^{{{\;}^{\circ }}^{\prime }}$ (i) the Ox form will behave as a 1-electron transferring species (*n* = 1) with an apparent *E*_m_ equal to $E_{\mathrm{Ox}/\mathrm{Sq}}^{{{\;}^{\circ }}^{\prime }}$; when $E_{\mathrm{Ox}/\mathrm{Sq}}^{{{\;}^{\circ }}^{\prime }}\ll E_{\mathrm{Sq}/\mathrm{Hq}}^{{{\;}^{\circ }}^{\prime }}$ (iii), the Ox form will behave as 2-electron transferring species (*n* = 2) with an apparent *E*_m_ similar to the actual one (0 mV). When $E_{\mathrm{Ox}/\mathrm{Sq}}^{{{\;}^{\circ }}^{\prime }}$ and $E_{\mathrm{Sq}/\mathrm{Hq}}^{{{\;}^{\circ }}^{\prime }}$ are less separated, with sufficiently precise experimental data it is possible to detect deviations from the theoretical behaviour of either a 1-electron or a 2-electron acceptor. This is depicted in panel (ii) for $E_{\mathrm{Ox}/\mathrm{Sq}}^{{{\;}^{\circ }}^{\prime }}=E_{\mathrm{Sq}/\mathrm{Hq}}^{{{\;}^{\circ }}^{\prime }}$. In this case, the apparent *E*°′ value of the Ox/Hq couple is higher than the actual one. (*d*) Shows the dependence of the Sq formation constant (*K*) and of the maximum concentration of Sq formed on the (*E*_1_−*E*_2_) difference of the standard redox potential values of the Ox/Sq (*E*_1_) and Sq/Hq (*E*_2_) couples. The two panels differ for the scale of the abscissa. The curves were drawn with the equations derived in the electronic supplementary material, section based on [29].

Flavins exist in the (yellow) Ox state and the (colourless) 2-electron fully reduced Hq form. The thermodynamic and/or kinetic stability of the 1-electron reduced Sq form varies broadly among flavoenzymes so that it may or may not be detected during equilibrium redox titrations or the catalytic cycle (figure 2). Two-electron transfer processes may be viewed as two consecutive 1-electron transfer steps in which, in the reduction direction, the redox potential of the Ox/Sq couple is much more negative than that of the Sq/Hq couple [29,30]. As a result, the stable flavin species are the Ox and fully reduced Hq ones, with the Sq going undetected because the second 1-electron transfer step is very fast. In other instances, the Sq does not form (e.g. when reduction takes place by hydride transfer from the substrate undergoing oxidation), or, in the case of 1-electron reduction, it is not detected because it rapidly equilibrates in an intermolecular disproportionation reaction of the type 2 Sq ↔ Ox + Hq, and the Ox and Hq forms prevail [29,31].

When the redox potential of the Ox/Sq couple is much less negative than that of the Sq/Hq one, the Sq is stabilized, and it may accumulate to different extents during a redox titration and/or the catalytic cycle. However, kinetic factors are also important for the stabilization of the flavin in its possible redox states. In flavodoxin (Fld), a small FMN-containing electron transfer flavoprotein, the neutral (blue) Sq is the stable species and Fld shuttles between the 2-electron reduced Hq and the Sq forms during processes in which it substitutes for ferredoxin (Fd) as a low potential electron donor in the −400 to −450 mV range under certain conditions (e.g. iron depletion). However, it is the FMN−protein interaction, rather than redox potential values, that avoids reaction with oxygen and Fld reoxidation making it a slow process. On the other hand, the electron transferring flavoprotein (ETF) shuttles between the Ox and the (anionic) Sq state of the bound FAD to mediate electron transfer from the several electron-donating flavin-dependent dehydrogenases to ETF : coenzyme Q (CoQ) oxidoreductase, another Fe-S flavoenzyme containing FAD and a [4Fe-4S] cluster [32]. Full ETF reduction to the Hq state is believed to be kinetically prevented by protein residues rather than to a much lower potential value of the Sq/Hq couple with respect to that of the Ox/Sq one. Indeed, *E*_m_ values of +4 mV for the Ox/Sq and −50 mV for the Sq/Hq couples have been reported for pig liver ETF [33].

The elective method to detect and characterize Ox, Hq and Sq forms of the flavin coenzymes is absorbance spectroscopy that exploits changes of the absorption spectrum in the visible region depending on the redox state, the protonation state of flavin dissociable positions, as well as changes in the flavin environment due to conformational changes or presence of ligands. Fluorescence spectroscopy can also help dissecting the reaction by exploiting the fluorescence of the Ox form that is lost upon reduction. However, flavin fluorescence is often quenched when protein bound limiting the application to, for example, quantifying the binding of the flavin coenzyme to the apoprotein or to monitoring protein denaturation [34]. Electron paramagnetic resonance (EPR) readily detects the Sq as an organic radical signal at *g* = 2 with characteristic temperature and power saturation behaviour. The linewidth of the saturable component allows to distinguish between the neutral (19 gauss) and the anionic form (15 gauss) [31,35,36]. Several volumes of the Methods in Molecular Biology series have been devoted to techniques employed in the flavoproteins field [37–39].

There seems to be no relation between the Sq stability, type (neutral or anionic), the reaction being catalysed and the overall protein (or protein domain) fold. Rather, fine details of the active site seem to modulate these properties. As an example, the ability of flavoenzymes of the oxidase class to reduce molecular oxygen to hydrogen peroxide, exemplified by mammalian D-amino acid oxidase, has been long associated with the ability to stabilize the red anionic Sq, and the formation of a covalent adduct with sulfite thanks to the presence of a positive charge near the N(1)-C(2) = O locus of the flavin [13]. However, in glutamate synthase (GltS), one of the Fe-S flavoenzymes that will be discussed below, the FMN coenzyme, bound to the (*α*/*β*)_8_ barrel domain of its *α* subunit, forms an adduct with sulfite and contains an *α* helix that points towards the N(1)-C(2) = O locus with its positive end, but the enzyme exhibits a low reactivity with molecular oxygen in agreement with the role of reduced FMN to reduce the 2-iminoglutarate (2-IG) intermediate to L-glutamate, most likely by hydride transfer from the FMN N(5) position [40]. Besides, no Sq is observed during redox titrations [41–44].

**Figure 3.**
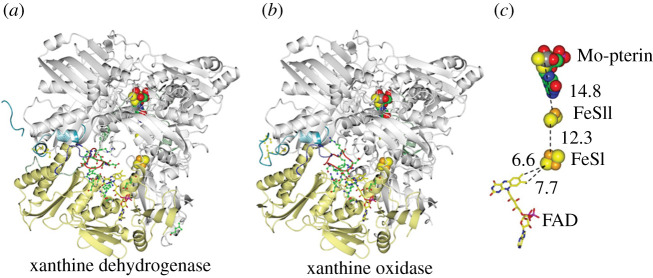
Comparison of the structures of the dehydrogenase and oxidase forms of bovine milk XOR. The conversion of xanthine dehydrogenase (XDH, (*a*), PDB ID 3UNC, [45]) into xanthine oxidase (XO, (*b*), PDB ID, 3AX9, [45]) brings along ordering of the C-terminal region (cyan), and both alteration of the electrostatics of the FAD environment and occlusion of the NAD^+^ binding site by repositioning of the 423–433 (red) loop, which interacts with the 494–504 (blue) loop, and of the region linking the Mo-pterin and FAD domains (residues 527–590, green). Cys residues, the oxidation of which mediates the conversion between the dehydrogenase and oxidase form, are indicated (when visible in the structures), namely Cys 992 and Cys 1317 for XDH, and Cys 992, 1317 and 1328 for XO. Several of the residues that are repositioned during the XDH to XO transition, which are believed to be important for the functional differences between the enzyme forms are shown as sticks [45–47]. (*c*) Shows the location and distances in angstroems between the redox centres. The distance between the Mo-pterin and the closest [2Fe-2S] cluster is calculated from the molybdenum atom.

A clear-cut example of the lack of relation between the fold, type of Sq and type of reaction is XOR, which will also be discussed in greater detail below. It exists in the oxidase (XO) or in the dehydrogenase (XDH) forms. XOR is formed by an N-terminal domain containing the two [2Fe-2S] clusters, the FAD-containing domain and a C-terminal molibdenum-pterin (Mo-pterin)-containing domain (figure 3). The prosthetic groups form a linear chain from the Mo-pterin, the spectroscopically distinguishable [2Fe-2S]^+1,+2^ clusters and FAD (which stabilizes the neutral Sq). Conversion of the native XDH to the XO form results from the reversible formation of key disulfide bridges or irreversible proteolytic events, which mainly alter the conformation of a few segments of the flavoprotein domain. In particular, conversion of XDH into XO removes from the active site a C-terminal peptide, which becomes exposed to solvent and disordered in XO. In the XDH/XO conversion, the active site geometry is also modified by an evident reorganization of the active site loop (Gln423-Lys433), as well as by more subtle changes. As a result of changes in the geometry of the active site and its electrostatics, binding of NAD^+^ is prevented in XO while maintaining the overall protein architecture [45–49].

## 3. The iron-sulfur clusters

**Figure 4.**
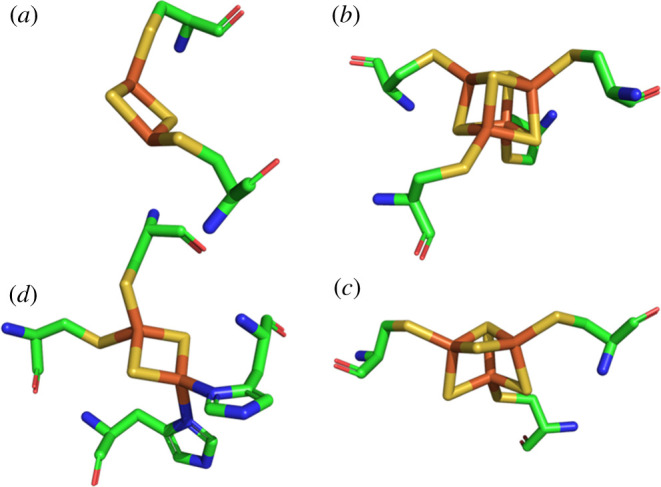
Fe-S clusters. Models of (*a*) [2Fe-2S], (*b*) [4Fe-4S] and (*c*) [3Fe-4S] clusters extracted from the crystal structure of *E. coli* SDH (PDB code: 1NEK [52]), and (*d*) of the Rieske-type centre extracted from the structure of mitochondrial complex III (PDB ID: 1SQV, [53]). The cysteinyl ligands (all clusters) and histidinyl ligands (Rieske-type cluster) are also shown. Colour code: iron, orange; sulfur, yellow; carbon, green; oxygen, red; nitrogen, blue. This and other figures including molecular models have been generated with CCP4 Molecular Graphics [54].

Iron-sulfur clusters are formed by iron ions, inorganic sulfur (sulfide anions) and protein side chains that anchor the cluster to the protein by binding to the iron ions [50,51]. They are believed to be the most ancient cofactors and include rubredoxin-type mononuclear clusters with no involvement of sulfide anions, [2Fe-2S], [3Fe-4S] and [4Fe-4S] clusters (figure 4), as well as extended clusters like siroheme in sulfite reductase, the P and MoFe clusters in nitrogenase, and the H cluster in hydrogenase [50]. Fe-S clusters typically accept/donate one electron shuttling between +2/+1 states (for the oxidized and reduced [2Fe-2S] and [4Fe-4S] clusters, respectively) or the +1/0 state (for the oxidized and reduced [3Fe-4S] centres, respectively). Textbook examples of Fe-S clusters are those found in Fds, small electron transfer proteins, the respiratory complexes I and III, as well as in succinate dehydrogenase (SDH, respiratory complex II) and the related FumR.

Absorbance and, especially, EPR spectroscopies are the elective techniques used to study Fe-S clusters. Other types of spectroscopy (e.g. Mossbauer and extended X-ray absorption fine structure (EXAFS) spectroscopies) are also very powerful tools to dissect their structure−function relations, while redox titrations and cyclic voltammetry provide information on the redox behaviour of the centres.

Besides being involved in electron transfer processes, Fe-S clusters are exploited in hydratase/dehydratase reactions, protein stabilization and signalling [50,55,56]. Aconitase is the prototype of enzymes using a [4Fe-4S] cluster to carry out a (de)hydration reaction. In the case of several enzymes acting on nucleic acids [57,58], the Fe-S and FAD-containing (6–4) photolyase form [59–61] or the related cryptochrome CryB [62], whether the [4Fe-4S] cluster only plays a structural role, or is also involved in electron transfer (in e.g. the Fe-S-containing photolyase) or catalysis (e.g. DNA primase) is less clear. That Fe-S clusters may have crucial structural roles in addition to their ‘classical’ electron transfer function was demonstrated, for example, in GltS. As discussed below, preventing the formation of the [4Fe-4S] clusters of the NADPH-dependent bacterial GltS, by site-directed mutagenesis of Cys ligands, abolished the association of the *α* and *β* subunits of the enzyme, and therefore the formation of the catalytically active *αβ* protomer [63].

Fds are the model Fe-S proteins and are found *per se* as electron transport proteins or as domains of complex proteins. They form a large class of proteins that differ in size, type and number of Fe-S clusters, partner proteins that donate/accept electrons, redox potential as modulated by the protein ligands and the overall protein environment [64,65]. Cysteine residues are the most common ligands of the iron atoms, but other residues are like Glu, Asp and His residues are often found. Their midpoint potential (*E*_m_) values vary from +80 to −700 mV, but are usually near −400 mV. The redox potential is modulated by H-bonds to the cluster, polarity of the cluster environment, and water access to the cluster, among others, but precise mechanisms are still incompletely understood. For example, contrary to expectations, the presence of an Asp ligand as one of the ligands of the [4Fe-4S] cluster of the *Pyrococcus furiosus* Fd is not associated with an anomalous redox potential value (approx. –350 mV) and the Asp14Cys or Asp14Ser substitutions had a moderate effect on the redox potential [*E*_m_ values −397 mV and −405 mV, for the D14C and D14S Fd variants, respectively [66].

## 4. Iron-sulfur flavoenzymes

**Figure 5.**
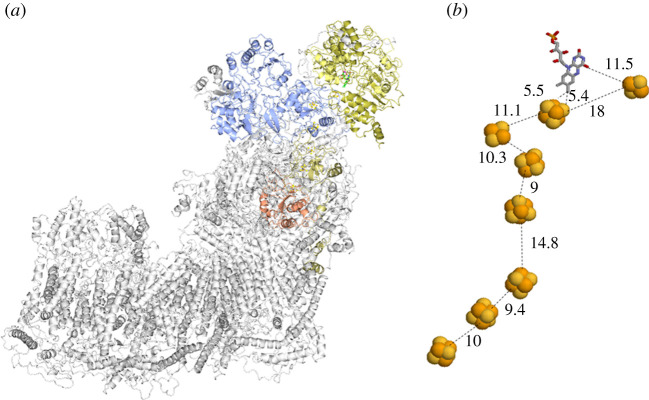
Structure of mammalian complex I. (*a*) The subunits of mouse complex I ((PDB ID: 6G2 J) [67]) are shown as ribbons. Only those containing redox centres are coloured. (*b*) Positions and distances (in Å) of the FMN coenzyme and of the Fe-S clusters.

**Figure 6.**
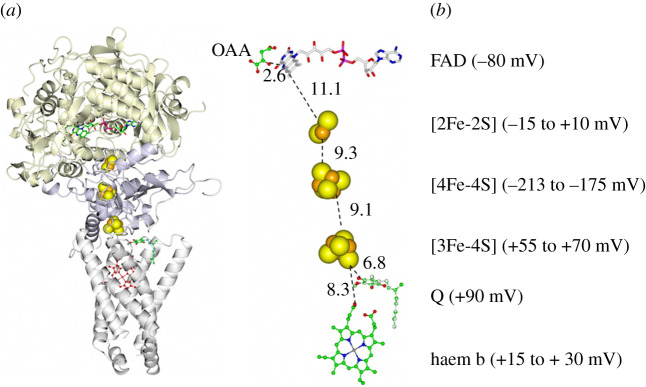
*Escherichia coli* SDH architecture. (*a*) The flavoprotein (gold), Fe-S (dark grey) and haem-containing (light grey) subunits of native *E. coli* SDH (PDB ID: 1 NEK) are shown with bound oxaloacetate (OAA) and ubiquinone (Q) [52]. (*b*) Geometry of the redox centres and ligands. Edge-to-edge distances are in Å. The redox potentials of the FAD coenzyme (as the Ox/Hq couple), the quinone acceptor (as the quinone/quinol couple), of the Fe-S centres (+2/+1 states for the [2Fe-2S] and the [4Fe-4S] centres and the haem b, and +1, 0 state for the [3Fe-4S] cluster) are indicated in parenthesis as reported in [74].

Well-known examples of Fe-S flavoenzymes are the respiratory complexes I and II. The L-shaped multi-subunit Complex I mediates the electron transfer from NADH to CoQ in the first step of the respiratory electron transfer chain with FMN acting as the electron entry site being reduced by hydride transfer from NADH. The chain of Fe-S clusters transports single electrons to CoQ across the membrane (figure 5). Up to 10 Fe-S clusters have been found in Complex I from different sources. In the core 14 subunits assembly of the *E. coli* protein (550 kDa), conserved from bacteria to mammals, six [4Fe-4S] centres and one [2Fe-2S] cluster form the internal electron transfer chain. An ‘off-chain’ additional [2Fe-2S] cluster near the FMN coenzyme may serve as electron storage/sink, may prevent reaction of reduced FMN with molecular oxygen limiting the harmful generation of reactive oxygen species and may serve a structural role or may just be an ‘evolutionary relict’. A second off-chain [4Fe-4S] cluster is not always present [67–70]. Indeed, respiratory Complex I is believed to derive evolutionarily from pre-existing modules. The multitude of Fe-S clusters in Complex I may reflect its modular evolution and has been proposed to be used to modulate electron transfer rates enabling synchronization with the much slower proton translocation rates [71]. How the oxidoreduction, taking place in the peripheral arm (7 subunits in *E. coli*), is coupled to the pumping of four protons for each oxidized NADH into the mitochondrial intermembrane space to promote ATP synthesis by the membrane arm has not been clarified yet, nor has the role of the additional subunits associated with the core ones [72]. However, it appears that proton pumping is not associated with electron transfer along the Fe-S cluster chain. Rather, the redox state of the quinone substrate has been suggested to be the key factor [73]. The Krebs cycle enzyme and respiratory complex II, succinate dehydrogenase (SDH), is a prototypical multi-subunit Fe-S flavoenzyme along with the closely related FumR that catalyses the reverse reaction. SDH uses FAD, which is covalently bound to the flavoprotein (*α*) subunit facing the mitochondrial matrix (in the eukaryotic form), to oxidize succinate with electrons being transferred to the second substrate (ubiquinone, CoQ) thanks to an electron transfer chain formed by three different Fe-S clusters: one [2Fe-2S]^+1,+2^ centre (proximal to FAD), one [4Fe-4S]^+1,+2^ cluster (in the middle of the subunit) and one [3Fe-4S]^0, +1^ centre (next to the CoQ binding site at the interface with the membrane) in the Fe-S (*β*) subunit (figure 6). Anchoring to the membrane is effected by two hydrophobic subunits (for *E. coli* and mammalian SDH). One of them harbours haem b in some, but not all, SDH (or FumR). Whether the latter is part of the electron transfer chain and may mediate electron transfer from the [3Fe-4S] cluster to CoQ, or the cluster directly reduces the quinone in two subsequent electron transfer steps is still not clear. However, its absence in several SDH suggests that it is not essential [75].

A [3Fe-4S] cluster resembles a [4Fe-4S] centres that has lost one of the iron ions (figure 4*c*) [51]. Indeed, when a [3Fe-4S] cluster is observed, establishing whether it is a native component of the protein, or a purification artefact is always an issue.

**Figure 7.**
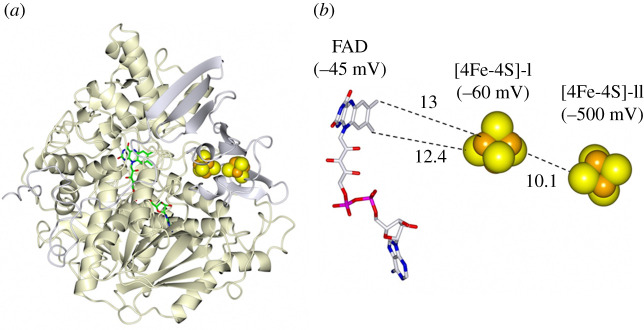
Structure and redox centres of *Archaeoglobus fulgidus* adenylsulfate (adenosine 5′-phosphosulfate, APS) reductase (PDB ID: 1JNR). (*a*) Ribbon representation of the 75 kDa flavoprotein *α* subunit (gold) and of the 20 kDa ferredoxin-like *β* subunit (grey). (*b*) The detail of the redox centres with distances in Å. The redox potentials of the FAD Ox/Hq, and Fe-S clusters in the +2/+1 state are indicated in parenthesis [76].

For example, in adenylsulfate (adenosine 5′-phosphosulfate, APS) reductase, another Fe-S flavoenzyme, which catalyses the reductive and reversible conversion of adenylsulfate into AMP and sulfite in sulfate-reducing and sulfide-oxidizing bacteria and archaea, one FAD was found on the *α* subunit, which is structurally related to the SDH/FumR flavoprotein subunit, while the *β* subunit was first reported to harbour one [4Fe-4S]^+1,+2^ cluster and one [3Fe-4S]^0,+1^ centre. However, it was later established that the active enzyme actually harbours two [4Fe-4S]^+1,+2^ clusters located at a close distance in the N-terminal Fd-like part of the *β* subunit (figure 7). Cluster I (−60 mV), at approximaely 12.5 Å from FAD, mediates the transfer of electrons from Cluster II (approx. −500 mV) close to the protein surface (where it should interact with an unknown electron donor) to FAD (*E*_m_, −45 mV for the Ox/Hq couple), which catalyses the reductive splitting of adenylsulfate to AMP and sulfite [76,77]. The large difference in redox potentials of the two clusters was explained by a larger number of interactions with polar residues of Cluster I with respect to Cluster II [76].

Aconitase is the prime example of an enzyme containing a [4Fe-4S]^+1,2^ cluster with a labile iron ion leading to an inactive enzyme containing a [3Fe-4S]^0,+1^ cluster. The enzyme uses the [4Fe-4S] cluster to catalyse the dehydration/rehydration reaction that converts citrate into isocitrate in the Krebs cycle. However, the labile iron ion was found to have an important regulatory role. The cytoplasmic form of aconitase doubles as an intracellular iron sensor, and in the [3Fe-4S]-containing form, it promotes iron uptake by directly binding and stabilizing the transferrin mRNA [50,55,56].

That [3Fe-4S] clusters could be native components of enzymes was demonstrated by observing, by EPR spectroscopy, its oxidoreduction directly in *E. coli* cells that had overproduced FumR, whose structure is closely related to that of SDH [78]. At temperatures below 30 K, and upon incubation with the oxidizing fumarate substrate, the anaerobically grown *E. coli* cells recovered the typical *g* = 2.016 sharp feature of the [3Fe-4S]^+1^ cluster that is detected in the purified protein. GltS have also been shown to be Fe-S flavoenzymes harbouring [3Fe-4S]^0,+1^ clusters [79–83]. That the [3Fe-4S] cluster is a native component of these enzymes was demonstrated with bacterial *Azospirillum brasilense* GltS [43]. The ‘as isolated’ bacterial NADPH-dependent GltS exhibited the EPR signature of one [3Fe-4S] cluster in the +1 state. At 5 K, an axial spectrum characterized by a sharp peak at *g*_ll_ = 2.03, and a broader g_⊥_, component at 1.97 was observed. That it was a native component of the enzyme was demonstrated by the ability of NADPH, the physiological enzyme reductant, to quantitatively reduce the cluster to the 0 state with the characteristic *g* = 12 signal, and subsequent full recovery of the [3Fe-4S]^+1^ signal upon enzyme reoxidation.

## 5. Directionality and rate of electron transfer processes

Determining the redox potential of prosthetic groups undergoing oxidoreduction is indeed key to the understanding of the reaction thermodynamics, thus the energy flux during a given process. However, it is also important to understand the rate at which such a process proceeds. Furthermore, in the case of proteins containing multiple redox centres, knowledge of the redox potential of each centre contributes to the determination of the direction of the electron transfer. In Fe-S flavoenzymes, the flavin coenzyme usually acts as a switch between the transfer of an electron pair (typically as a hydride anion from/to a reduced pyridine nucleotide or an organic substrate), and the transfer of single electrons to one or more Fe-S clusters that may form a clear linear electron transfer chain, or may be placed at positions that are consistent with multiple electron transfer paths. Hydride transfer in flavoenzymes typically requires a donor/acceptor distance of the hydrogen atom of approximately 3.5 Å [26]. According to theory and experimental observations ([84,85] and references therein), due to their small mass, electrons tunnel through the energy barrier separating reactants from products, and their behaviour is well described by taking into account their wave-particle duality. As summarized by Moser *et al.* [85], the rate of electron transfer depends on the energy difference between products and reactants (the free energy change under standard conditions, Δ*G*°, and therefore the corresponding redox potential difference), the reorganization energy (*λ*, i.e. the energy difference between products and reactants accounting for (nuclear) reorganization of the molecule without the actual electron transfer), and the electronic coupling between the initial and final state. The latter is proportional to the overlap of the donor and acceptor wave functions across the space separating electron donor and acceptor molecules. Such overlap decreases exponentially as the edge-to-edge distance (*R*) between the molecules increases and is affected by the intervening medium. A simplified semi-empirical expression for the calculation of the rate of electron transfer in biological systems is shown in eq. (5.1) for an exoergonic reaction, and in eq. (5.2) for an endoergonic reaction.

5.1 $$\log_{10}k_{\mathrm{et}}^{\mathrm{ex}}=13-0.6(R-3.6)-3.1{\left(\Delta G^{o}+\lambda \;\right)}^{2}/\lambda$$

and

5.2 $$\begin{array}{r} \log_{10}k_{\mathrm{et}}^{\mathrm{en}}=13-0.6(R-3.6)-3.1{\left(\Delta G^{o}+\;\lambda \right)}^{2}/-\;\Delta G^{o}/0.06 \end{array}.$$

In these equations, which report on the value of the rate constant of electron transfer for an exoergonic $\left(k_{\mathrm{et}}^{\mathrm{ex}}\right)$ or endoergonic $\left(k_{\mathrm{et}}^{\mathrm{en}}\right)$ reaction on a decimal log scale, 13 expresses the maximum electron transfer rate that can be achieved when the donor–acceptor pair is at van der Walls distance. Indeed, 10^13^ s^−1^ corresponds to kT/h preexponential factor of the expression of the rate of a chemical reaction according to the classical transition state theory when no activation energy barrier needs to be overcome and the transmission coefficient is 1 and corresponds to the vibrational rate; 0.6 takes into account the effect of the medium separating the donor–acceptor couple on the electronic coupling. The latter is expressed as the difference between the edge-to-edge distance of the donor/acceptor pair (R in Å) and their minimal distance, i.e. their van der Walls contact distance (3.6 Å). ΔG° (the equilibrium energy difference between donor and acceptor) and the reorganizational energy *λ* are in electronVolts (1 eV = 1.6 × 10^–19^ *J* = 3.8 × 10^–20^ cal, i.e. 23.06035 kcal mol^−1^ taking into account the Avogadro's number: 6.0221367 × 10^23^ mol^−1^). In equation (5.2), the rate constant for an endoergonic electron transfer step can be calculated from the rate of the corresponding reverse (exoergonic) reaction and a penalty of –Δ*G*°/0.06. Thus, the electron transfer of an uphill reaction will slow down by a factor of 10 for every 0.06 eV (approx. 1.4 kcal mol^−1^) of uphill Δ*G*°, as compared to the reverse (thermodynamically downhill) reaction.

It has been calculated that electron transfer can take place at rates as high as 10^7^–10^13^ s^−1^ when donor–acceptor couples are separated by up to 14 Å in Δ*G* optimized electron transfer (Δ*G*° = −*λ*). At shorter distances, thermodynamically uphill steps can take place very rapidly. It has been calculated that at a 6 Å distance, tunnelling rate is maximal at 3 × 10^11^ s^−1^ when Δ*G*° matches −*λ*, remains high at 2 × 10^8^ s^−1^ when Δ*G*° = 0, and is still 10^2^ s^−1^ for a +0.5 eV (approx. 11.5 kcal mol^−1^) endergonic step [85,86].

Overall, electron transfer in electron transfer chains is usually much faster than steps involving group transfer, such as hydride transfer in NAD(P)H oxidation, or proton transfer, so that reactions involving electron transfer chains formed by Fe-S clusters in Fe-S flavoenzymes are usually limited by the hydride transfer step from the reducing substrate. The high rate of electron transfer along with the need of rapid reaction techniques able to distinguish among redox centres, and to precisely quantify them (e.g. the already mentioned EPR spectroscopy coupled to continuous flow/freeze-quench methods) make their experimental determination difficult. As an alternative the slow step may be a protonation step or a conformational change that would bring the electron donor/acceptor pair at a distance sufficiently close to allow for electron transfer [85,86].

**Figure 8.**
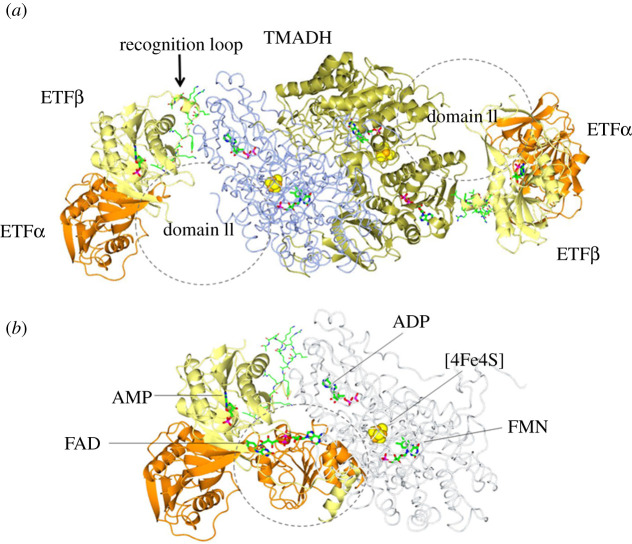
Structure of the ETF in complex with TMADH. (*a*) The high-resolution structure of *Methylophilus methylotrophus* ETF-TMADH complex (1O94) [87] shows the TMADH homodimer (in gold and ice blue) with one molecule of ETF bound to each subunit (*α* subunit, orange; *β* subunit, lemon). ETF domain II, the FAD-containing domain mainly formed by *α* subunit, is not visible in the crystal structure indicating that it is highly mobile. Its proposed approximate position is indicated with a circle. (*b*) Shows the superposition of the complete ETF structure (1O97) [87] with that of one ETF copy (colour code as in the (*a*)) and the interacting TMADH subunit (now in worms and light grey). Domain II nicely occupies the hypothesized position. However, for productive electron transfer from the [4Fe-4S] cluster (spacefill), this domain must be endowed of a large degree of conformational flexibility in order to bind to TMADH orienting the FAD isoalloxazine ring towards the Fe-S cluster. The molecular dynamics calculations reported by [87] show that this is possible.

The latter mechanism is exemplified by the already mentioned ETF. This FAD-containing electron transfer protein is characterized by high conformational flexibility, which allows it to interact with its several electron donor protein partners and ETF : CoQ oxidoreductase. The high-resolution crystallographic structure *Methylophilus methylotrophus* ETF-trimethylamine dehydrogenase (TMADH) complex clearly showed a bimodal binding mode (figure 8). Domain III (formed by the majority of the ETF *β* subunit) serves to anchor ETF to TMADH. It also allows the FAD-containing domain II of ETF (the C terminus of *α* subunit and a small C-terminal region of *β* subunit) to probe different conformations and, eventually, properly interact with TMADH to allow for electron transfer. In agreement with the shuttling of ETF between the Ox/Sq states, TMADH is a Fe-S flavoenzyme containing one FAD (at the trimethylamine oxidizing site) and one [4Fe-4S] centre, the latter being the site of TMADH-to-ETF electron transfer through the likely intermediacy of a tyrosine residue [87]. Such mechanism is reminiscent of the conformational changes that give rise to the Q cycle in respiratory complex III, which uses a Rieske-type [2Fe-2S] cluster to mediate transfer of electrons from CoQ to cytochrome c via cytochrome c_1_, as well as to haem b_L_ and b_H_. The mobility of the Fe-S-containing subunit of Complex III is key to the onset of the Q cycle that exploits the stability of the Sq form of CoQ to set-up a bifurcated electron transfer pathway, and therefore the reaction mechanism that, eventually, guarantees that four protons are transferred to the mitochondrial intermembrane space for each CoQ being oxidized [88,89].

**Figure 9.**
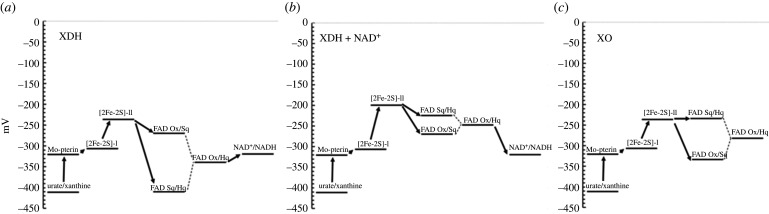
Scheme of the redox potential values of the dehydrogenase and oxidase forms of XOR, and effect of the NAD^+^ substrate. The midpoint potential values of the redox centres of xanthine dehydrogenase (XDH) in the (*a*) absence and (*b*) presence of NAD^+^, and (*c*) of XO as reported in [45,90]. The electron transfer to molecular oxygen from FAD in XO is not shown being a strongly exoergonic step.

A well-recognized complication to the understanding of the redox properties of a given enzyme and, thus, to the understanding of the molecular bases of electron transfer direction and rates, resides in the sensitivity of the potential of redox centres to their microenvironment. Therefore, the presence of substrates and reaction intermediates, changes in protonation of groups, and small conformational changes that take place during the catalytic cycle may dramatically (and transiently) alter the redox potential of the cofactors, which is usually studied under equilibrium conditions. In the already mentioned, well-characterized, XOR, the conformational changes associated with the XDH/XO transition not only prevent NAD^+^ binding, but also alter the FAD redox properties with little or no effects on those of the Mo-pterin cofactor and of the two [2Fe-2S] clusters as nicely reviewed in [90]. In particular, in XDH the standard redox potential of the Sq/Hq couple (−410 mV) is much lower than that of the Ox/Sq pair (−270 mV). While electron transfer from the Fe-S cluster closer to the flavin (Fe/S-II, −235 mV) would allow transfer of the first electron to generate the flavin Sq, full reduction to the Hq form would be an approximately 175 mV thermodynamically uphill process. The conversion of XDH to the XO form dramatically (and specifically) destabilizes the Sq by approximately 180 mV bringing the midpoint potential value of the Sq/Hq couple to −234 mV, well above that of the Ox/Sq couple (−332 mV) and close to that of the Fe/S-II (−235 mV) (figures 3 and 9). Computational work, which exploited the high-resolution structures of XDH in the free, NAD^+^ and NADH-bound state and of a protein form locked in the XO conformation, allowed to dissect the (often opposite) contributions of ‘protein hydrophobicity’, electrostatics, neighbouring Fe-S clusters and Mo-pterin on the redox properties of the FAD coenzyme [45]. Furthermore, the study revealed—at the atomistic level—how NAD^+^ binding to the enzyme during the catalytic cycle has a dramatic effect on the redox potential of the FAD Sq/Hq couple that reaches a value of −226 mV, similar to that of the same species in the XO form, with no significant effect on the redox potential of the Ox/Sq couple, and a modest effect on that of the [2Fe-2S] centre (−200 mV). Overall, the NAD^+^ substrate makes the two subsequent 1-electron transfer steps from Fe/S-II less thermodynamically unfavoured by only 70 mV (first electron transfer to convert the flavin from the Ox to the Sq state) and 26 mV for the second electron transfer step to convert the flavin Sq to the Hq. The redox potential of the urate/xanthine couple is very low (−410 mV at pH 7.65 and −441 mV at pH 8.07 [29]). Thus, the overall oxidoreduction is thermodynamically favoured by an approx. 90 mV difference between the xanthine/urate and NAD^+^/NADH couples (−320 mV under standard conditions). Furthermore, the low potential of the xanthine/urate couple fully justifies the favoured electron transfer to the Mo-pterin cofactor (−320 mV) and from this to the closest [2Fe-2S] cluster (Fe/S-I, −310 mV) and that closest to FAD (Fe/S-II, −235 mV in the free XDH and XO, or −200 mV in the XDH/NAD^+^ complex). However, the increase of the midpoint potential of the FAD Sq/Hq couple upon NAD^+^ binding, while favouring the formation of the flavin Hq by FeS-II, also increases the midpoint potential of the FAD Ox/Hq couple from −340 (free XDH) to –248 mV (similar to that calculated for FAD Ox/Hq couple in XO, −283 mV) leaving NAD^+^ reduction as a thermodynamically uphill step. How protein dynamics may alter the above scheme still needs to be established in this and other systems. Biologically, the balance of redox potential values of the various prosthetic groups and final electron acceptor in XDH may avoid over-reduction of the enzyme, which just needs two electrons being transferred from xanthine to NAD^+^, while a total of 5 may be loaded onto the enzyme.

## 6. Iron-sulfur flavoproteins with diverse functions, but a common module

The prototype of a flavoenzyme able to interact with an electron transferring Fe-S protein is Fd : NADP^+^ oxidoreductase (FNR) found in the terminal step of the photosynthetic electron transfer chain. Here, the FAD-dependent enzyme plays a classical role by coupling two electrons from two reduced Fd molecules and transferring the electron pair, as a hydride anion, to NADP^+^ generating NADPH. Therefore, FNR makes reducing the power available for CO_2_ fixation, as well as other reactions. Why FNR does not react with Photosystem I, being directly reduced in two subsequent electron transfer steps through the flavin Sq, probably depends on evolution, which led first to the introduction of Fd as the terminal electron acceptor in the photosynthetic electron transfer chain, and as the electron donor to a variety of Fd-dependent biosynthetic enzymes. Several FNR act in the opposite direction by oxidizing NAD(P)H in order to supply reduced Fd for such biosyntheses. Here the role of the Sq as a provider of electrons at a potential sufficiently low to generate reduced Fd is evident, making it possible to use NAD(P)H (*E*_m_, approx. −320 mV) to reduce Fd (*E*_m_, −400 mV). NAD(P)H-reduced FAD stabilizes the blue Sq, thus the potential of the Sq/Hq couple is lower than that of the Ox/Sq couple making it possible to donate one electron to Fd at a low potential. Disproportionation reactions, which imply intermolecular electron transfer between FNR molecules, ensure the equilibration between Sq, Ox and Hq forms [64,91].

Gene fusion events may have generated several Fe-S flavoproteins by linking flavoprotein and Fd modules. For example, phthalate dioxygenase reductase (PDR), a well-characterized Fe-S flavoenzyme, appears to be formed by an FNR-like module (although it binds FMN rather than FAD) and a [2Fe-2S] plant-type Fd [92].

More complex events may have generated the examples of Fe-S flavoenzymes mentioned before, namely xanthine oxidase/dehydrogenase, respiratory complexes I and II and other instances.

In the respiratory Complex I and the Krebs cycle enzyme SDH (figures 5 and 6), the flavin is at the electron entry site (FMN for Complex I and the covalently bound FAD for SDH) and accepts the electron pair, as a hydride anion, from NADH (Complex I) or succinate (SDH). The electron transfer chain formed by the Fe-S clusters connects the flavin and CoQ, the second reaction substrate, which can also stabilize a Sq. In principle, a direct oxidoreduction between the flavin and the quinone substrate would be possible, but the intramolecular electron transfer chain formed by the Fe-S clusters may be needed to make NADH (or succinate) oxidation irreversible (or favoured), to guarantee the directionality of the process (and coupled proton transfer in Complex I) across the membrane, and to avoid (or at least limit) the generation of reactive oxygen species. Indeed, in these cases, the flavin is reduced by hydride transfer, and it is rapidly reoxidized as the electrons are rapidly transferred to the Fe-S clusters that act as temporary electron sinks for the reducing equivalents. Thus, the reaction of the reduced flavin with oxygen is avoided, and further NADH (or succinate) oxidation is promoted. The distribution of electrons among the Fe-S clusters may also similarly control CoQ reduction.

Gene duplication and evolution with adaptation to the metabolic properties of a given organism may have led to the use of a similar module in otherwise unrelated enzymes. This concept will be exemplified below by discussing bacterial GltS, mammalian dihydropyridine dehydrogenase (DPD) and archaeal NADH-dependent reduced Fd : NADP^+^ oxidoreductase (Nfn). These enzymes share a subunit or domain that makes reducing equivalents from reduced pyridine nucleotides available to other unrelated subunits (or domains) through low to very low potential [4Fe-4S]^+1,+2^ clusters giving rise, in the case of *P. furiosus* NfnI and the corresponding *Thermothoga maritima* NfnAB, to the newly discovered FBEB mechanism of energy conservation. Such conserved common subunit (or domain) is built of an adrenodoxin reductase-like domain, containing both the FAD coenzyme and the NAD(P)H binding site, preceded by an N-terminal domain forming two low potential [4Fe-4S]^+1,+2^ clusters. It will be named GltD-like from the gene name of the corresponding small (*β*) subunit of bacterial GltS. Indeed, it was starting from the GltS *β* subunit (GltD) primary structure that a series of proteins containing GltD-like subunits or domains were identified [93]. They included, besides NAD(P)H-dependent GltS forms and mammalian DPD, the large subunit of *P. furiosus* sulfide dehydrogenase [94], which has now been identified as NfnI [95], and the C-terminal domain of *E. coli* AegA.

### 6.1. Glutamate synthase

GltS may serve to exemplify features that characterize several other complex Fe-S flavoenzymes with respect to their initial assembly, during evolution, from unrelated modules. The coevolution of such modules eventually led their intimate connection in the resulting protein and can no longer be viewed as individual entities. GltS is essential for ammonia assimilation processes in microorganisms and photosynthetic cells. The enzyme catalyses the reductive synthesis of L-glutamate from 2-oxoglutarate (2-OG) and L-glutamine and forms with glutamine synthetase a key pathway for ammonia assimilation, especially when free ammonia levels are low (in microorganisms) or during photorespiration to avoid the toxicity of the released ammonia (in plants).

**Figure 10.**
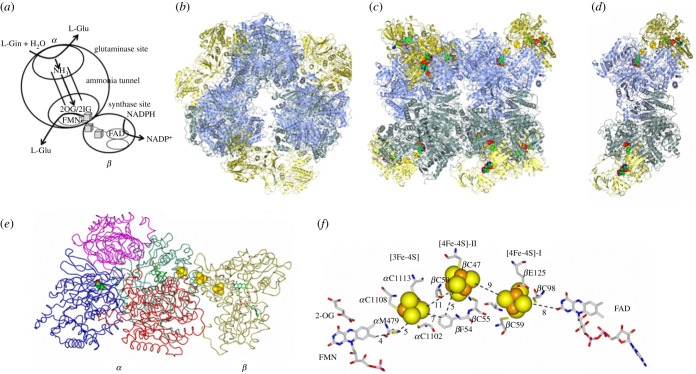
Structure of the 1.2 MDa NADPH-dependent GltS (*αβ*)_6_ hexamer. (*a*) Scheme of the partial activities associated with the catalytic subsites of bacterial GltS. The cryo-electron microscopy-derived model of NADPH-dependent GltS at 4 Å resolution (PDB ID: 6S6X [96]) confirmed and refined the previous 9.4 Å model (PDB ID: 2VDC [97]) showing that GltS forms a 1.2 MDa complex ((*b*), top view; (*c*), side view) formed by the assembly of three (*αβ*)_2_ pillars (*d*). The pillars reflect the crystallographic dimer of *α* subunit [40] with *β* subunits attached at the periphery to yield the catalytically active *αβ* protomer (*e*). In (*c*, *d*): the *α* subunits are ice blue (top layer) or grey-blue (lower layer); the *β* subunits are gold (top layer) or lemon (lower layer); the prosthetic groups are in spacefill. The catalytically active *αβ* protomer extracted from the side view (*c*) is shown in (*e*). Here, the *β* subunit is in gold; the domains of the *α* subunit are colour coded as follows: glutaminase domain (residues 1–422), blue; central domain (423–780), red; synthase domain (781–1202), green; C-terminal β-helix (1203–1472), purple. Cys1 of the *α* subunit is shown in spacefill to mark the glutaminase site. FAD, FMN and 2-OG are in sticks; the Fe-S centres in spacefill. (*f*) Shows the chain formed by the five prosthetic groups of the enzyme, the ligands to the Fe-S centres and distances in angstroems. The *α* subunit Met 479, which is the only residue falling in a disallowed region of the Ramachandran plot, may control (or even participate in) electron transfer between the [3Fe-4S] cluster and FMN. The *β* subunit Phe 54 may participate in electron transfer between [4Fe-4S]-II and the [3Fe-4S] centre.

Bacteria contain a NADPH-dependent GltS (NADPH-GltS) formed by two subunits (α-GltS, encoded by *glt*B gene, approx. 150 kDa; β-GltS, encoded by *glt*D gene, approx. 50 kDa) harbouring five different prosthetic groups (one FAD, one FMN, one [3Fe-4S]^0,+1^ centre and two [4Fe-4S]^+1,+2^ clusters; figure 10). During the reaction, L-Gln binds to the PurF-type glutaminase domain on α-GltS. Here, L-Gln is hydrolysed to yield L-Glu and ammonia. The latter diffuses through an approximately 30 Å-long intramolecular ammonia tunnel to reach the synthase site where it adds to 2-OG bound in front of the FMN coenzyme to yield 2-IG. The intermediate is reduced by hydride transfer from reduced FMN to yield L-Glu. FMN is reduced to the Hq form by electrons deriving from NADPH. The pyridine nucleotide substrate binds and reduces FAD on the *β* subunit (β-GltS). The latter has an adrenodoxin reductase-like domain and an N-terminal extension, which contains the ligands to the two [4Fe-4S]^+1,+2^ clusters of NADPH-GltS that form, with the [3Fe-4S]^0,+1^ cluster (on α-GltS), the intramolecular electron transfer chain connecting the electron entry site (FAD on β-GltS) to the electron exit site (FMN on *α*GltS). In photosynthetic bacteria and plant chloroplasts, a Fd-dependent GltS form exists (Fd-GltS). The enzyme is similar in size, cofactor content (one FMN and one [3Fe-4S] cluster), structure and function (glutamine hydrolysis, ammonia transfer and addition to 2-OG, reductive L-Glu synthesis from 2-IG) to the bacterial α-GltS, but the reducing equivalents are acquired through reversible association with reduced Fd. In eukaryotic microorganisms and lower animals, the two bacterial *α* and *β* subunits are fused together to yield a single long polypeptide, and the electron donor is NADH instead of NADPH. Interestingly, the photosynthetic bacterium *Synechocystis* sp. PCC6803 contains one gene encoding an Fd-GltS and genes encoding the *α* and *β* subunits of a pyridine nucleotide-dependent bacterial GltS, which, however, appears to be NADH-dependent. Such genes do not form the typical bacterial operon for GltS and may represent an intermediate evolutionary stage between bacteria and eukaryotes. Open-reading frames potentially encoding individual domains of the NADPH- and Fd-dependent GltS have been identified in Archaea, suggesting an ancient origin of the protein (reviewed in [93,98–102]). Interestingly, ammonia tunnels connecting the glutaminase and synthase domains are a typical feature of the amidotransferase class of enzymes [103,104]. They represent an example of convergent evolution being formed by the unrelated synthase sites rather than the conserved amidotransferase domains. In GltS, which is the only amidotransferase exploiting oxidoreduction to drive ammonia addition to the accepting molecule, the tunnel is lined by residues of the FMN/synthase domain and the so-called central domain. The latter is a truncated *α*/*β* barrel, which may derive from duplication of the (*α*/*β*)_8_ barrel forming the FMN/synthase domain with the loss of secondary structure elements, or have an independent origin [40].

The association of the NADPH-GltS *α* subunit with the *β* subunit couples glutamine hydrolysis to ammonia transfer and 2-IG formation and reduction within *α* subunit [44]. The presence of 2-OG and reducing equivalents are also required to activate the glutaminase site across the ammonia tunnel so that wasteful glutamine hydrolysis is avoided. The association of *α* and *β* GltS subunits is also necessary in order to incorporate the [4Fe-4S] clusters into the N-terminal region of β-GltS [105]. On the other hand, the integrity of both the [4Fe-4S] clusters in the N-terminal region of β-GltS is required for the formation of the *αβ* protomer, as they ensure the correct conformation of the interface region between the subunits [63]. These features may be shared by other Fe-S flavoenzymes where the Fe-S cluster(s) carrying domain is strategically located at the interface between domains or subunits ensuring electron transfer between redox centres.

The β-GltS [4Fe-4S]^+1,+2^ clusters exhibit low to very low redox potentials being only marginally reduced by NADPH, which, however, readily reduces the [3Fe-4S]^0,+1^ cluster on *α*GltS, or dithionite. One of the [4Fe-4S] clusters is quantitatively reduced by NADPH in the presence of a NADPH-regenerating system formed by glucose 6-phosphate and glucose 6-phosphate dehydrogenase. Both are reduced photochemically, thus by inputting electrons at a low potential [43]. That both the [4Fe-4S] clusters participate in electron transfer from FAD to FMN has been first hypothesized, and later confirmed and clarified by determining the NADPH-GltS structure by cryo-electron microscopy (figure 10) [96,97].

**Figure 11.**
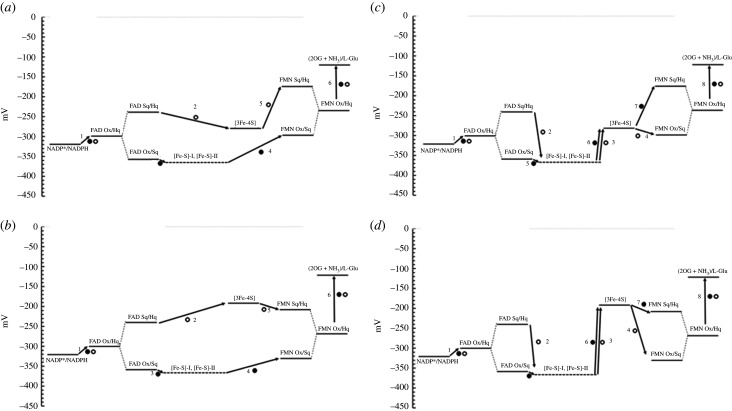
Proposed electron transfer pathways for NADPH-dependent GltS. (*a*,*b*) Bifurcated electron transfer from FAD to the Fe-S clusters and confurcation on FMN in the absence (*a*) and presence of 2OG(*b*). (*c*,*d*) Linear electron transfer pathway from FAD to FMN through the linear chain formed by the GltS Fe-S clusters. The redox potential values of the [4Fe-4S] clusters have not been determined, but they have been assumed to be similar or lower than that of the FAD Ox/Sq couple and are indicated with a dashed line. Numbers indicate the sequence of steps of the first (empty circle) and second (full circle) leaving FAD [41].

The measurement of the redox potential values of the FAD and FMN coenzymes and of the [3Fe-4S] cluster of NADPH-GltS could be done by carrying out absorbance-monitored redox titrations thanks to the differences in their redox potential values [41]. No Sq formation was observed during titrations setting limits for the redox potentials of the FAD and FMN Ox/Sq and Sq/Hq couples, which must be separated by at least 120 mV. With the assumption that both [4Fe-4S] clusters (with potential as low or lower than that of the FAD Ox/Sq couple) were involved, and in the absence of structural information on the GltS *αβ* protomer, the data allowed to propose two schemes for electron transfer from FAD to FMN (figure 11) [41].

In one scheme, the electron transfer from FAD to FMN would follow a bifurcated (and then confurcated) pathway, which would fully exploit the low redox potential of the FAD Ox/Sq couple to promote the reduction of the low potential [4Fe-4S] clusters with NADPH as the reductant. In such a scheme, the two electrons loaded on FAD would follow two different pathways on their way to FMN. The first electron (*E*_m_ for FAD Sq/Hq couple set at −240 mV from an experimentally determined *E*_m_ value of −300 mV for the FAD Ox/Hq couple and separation of the potential of the Ox/Sq and Sq/Hq couples of at least 120 mV) could directly reduce the [3Fe-4S] cluster (*E*_m_, −280 mV) leaving the second electron at a potential sufficiently low (estimated *E*_m_, −360 mV for the FAD Ox/Sq couple) to reduce the [4Fe-4S] centres (at an estimated potential similar or lower than that of the FAD Ox/Sq couple, ≤360 mV). An electron from these centres would have a potential sufficiently low to reduce FMN to the Sq form (*E*_m_ of the FMN Ox/Sq couple estimated to be approximately −300 mV from the experimental *E*_m_ of the FMN Ox/Hq couple of −240 mV, and a separation of the Ox/Sq and Sq/Hq couples of at least 120 mV) making electron transfer from the [3Fe-4S] cluster (−280 mV) to FMN Sq (estimated *E*_m_ for the FMN Sq/Hq couple, −180 mV) possible. The alternative proposed scheme implied a linear electron transfer pathway from FAD to FMN through the [4Fe-4S] centres and the [3Fe-4S] cluster. It also exploited the low potential of the Ox/Sq couples for both FAD and FMN, but required a greater number of thermodynamically uphill steps. In this case, the first electron from reduced FAD (−300 mV) would be transferred to one of the low potential [4Fe-4S] centres (less than or equal to −360 mV) in a thermodynamically uphill step, which would be promoted by thermodynamically downhill transfer to the [3Fe-4S] cluster (−280 mV) of the same electron and of that of the second one. Electron transfer from the reduced [3Fe-4S] centre to the oxidized FMN (−300 mV) would also be thermodynamically unfavoured, but the completion of the reduction of FMN with the conversion of the Sq to the Hq form (−180 mV) would be a thermodynamically downhill step. Interestingly, the association of the two subunits afforded a 40 mV increase of the *E*_m_ value of the FAD Ox/Hq couple with no significant change of those of the FMN and [3Fe-4S] clusters. Among the enzyme substrates, 2-OG increased the potential of the [3Fe-4S] cluster (to –190 mV) and lowered that of FMN (*E*_m_ of the Ox/Hq couple to −270 mV leading to the estimate of *E*_m_ values for the Ox/Sq and Sq/Hq couples of −330 mV and –210 mV, respectively), reducing the number of thermodynamically uphill steps in the bifurcated pathway, but not in the linear one. Establishing which electron transfer pathway is operative in NADPH-GltS had to wait until a structural model of the 1.2 MDa NADPH-GltS (*αβ*)_6_ oligomer was obtained by cryo-electron microscopy at 9.4 Å resolution first [97] and approximately 4 Å resolution, more recently [96].

Both models clearly showed that the electron transfer pathway from FAD and FMN is linear with the two [4Fe-4S] centres and the [3Fe-4S] cluster forming an arc joining FAD and FMN (figure 10). Protein residues (namely Phe 54 of *β* subunit and Met479 of the *α* subunit) may mediate electron transfer between centres (figure 10).

Another interesting feature of GltS consists of observations that strongly suggest that the enzyme shuttles between the 2-electron and the 4-electron reduced form during the catalytic cycle and undergoes a series of priming steps. In NADPH-GltS, absorbance and EPR-monitored experiments showed that NADPH reduction leads to a species in which one flavin is fully reduced along with the [3Fe-4S] cluster, and one reducing equivalent seems shared by a flavin (neutral) Sq and one of the [4Fe-4S] cluster [43]. For Fd-GltS, which is monomeric and forms a 1 : 1 complex with Fd [106], at least in the oxidized state, the activating effect of reduced Fd in complex with the fully (3-electron) reduced Fd-GltS on the L-Gln-dependent L-Glu synthesis led to propose that Fd-GltS undergoes three subsequent priming steps by receiving three electrons from reduced Fd (one at a time). Then, when in a complex with one molecule of reduced Fd, it can carry out one catalytic cycle. Here, reduced FMN is the direct hydride donor to the 2-IG intermediate, but the oxidized coenzyme is rapidly refilled with electrons at the expenses of the reduced [3Fe-4S] cluster and the bound reduced Fd. Now one priming step and association with a reduced Fd molecule would be sufficient to lead to the catalytically competent species [42,99].

Thus, in GltS the modular structure of the enzyme allows to carry out an essential reaction by adapting to the cell metabolism, while the ‘cross-talk’ of different modules of the NADPH-GltS *αβ* assembly or Fd/Fd-GltS complex ensures efficiency and regulation of the reaction.

Protein subunits or domains remarkably similar to the NADPH-GltS *β* subunit have been found by early sequence comparisons [93]. They were proposed to form a class of β-like (or GltD-like, from the gene name) proteins that serve to make the reducing equivalents of NAD(P)H available to a second protein or protein domain through low to very low [4Fe-4S] clusters encoded by the GltD-like N-terminal domain and interface region.

### 6.2. Dihydropyrimidine dehydrogenase

**Figure 12.**
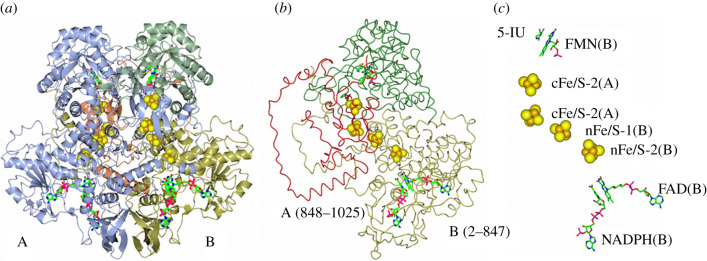
Structure and electron transfer pathway in mammalian DPD. (*a*) The catalytically active DPD dimer in complex with NADPH and 5-iodouracil (5IU, PDB ID: 1GTH [109]) with subunit A in ice blue and subunit B domains coloured as follows: GltD-like domain (residues 2–524), gold; the pyrimidine reducing dihydroorotate reductase-like site (residues 525–847), green; the C-terminal Fe-S containing domain (residues 848–1025), coral. The four [4Fe-4S] clusters are shown in spacefill; the FAD and FMN coenzymes, and the 5IU and NADPH ligands are in sticks. (*b*) The 2–847 region of the B subunit is in worm representation and colour coded as in panel (*a*); the C-terminal domain of subunit A (residues 848–1025) is in red to show how the C-terminal [4Fe-4S] clusters complete the electron transfer chain from FAD to FMN, via the N-terminal [4Fe-4S] clusters, on the other subunit. (*c*) Shows the location of the redox centres and ligands and their distances. The labels include the subunit to which the ligands belong. Distances are indicated in Å.

A GltD-like domain is found in mammalian dihydropyrimidine dehydrogenase (DPD). The overall reaction consists of the NADPH-dependent reduction of uracil to dihydrouracil in the catabolism of pyrimidines. Since also 5-fluorouracil is a good substrate, DPD inhibitors are being searched for to be used in anti-cancer therapy with 5-fluorouracil in order to both lower the amounts of drug to be administered, and to decrease the toxicity of its breakdown products through the reaction initiated by DPD. Furthermore, patients carrying genetic defects that lower their DPD activity require adjustment of 5-fluorouracil therapy to avoid over dosage [107,108]. In DPD, the homodimer (2 × 111 kDa) is the active form. Each subunit is formed by an N-terminal GltD-like domain harbouring one FAD (the electron entry site from NADPH) and two [4Fe-4S] clusters, an FMN-containing DHODH-like domain and a C-terminal extension similar to 8-iron Fds hosting two [4Fe-4S] centres (figure 12) [107,109,110].

In the homodimer, the GltD-like N-terminal region of one subunit, harbouring two [4Fe-4S] clusters (named nFeS1 and nFeS2 in DPD), makes contacts with the C-terminal 8Fe Fd-like domain of the other chain that carries another two [4Fe-4S]^+1,+2^ clusters (named cFeS1 and cFeS2). The linear chain formed by such four clusters connects FAD at the NADPH oxidizing site and FMN at the uracil reducing site. A peculiar feature of DPD is that, in such apparently simple intramolecular electron transfer chain, the electrons pass from one subunit to the other in the functional heterodimer twice, namely: from FAD to nFeS2 and then nFeS1 (of one subunit) to cFeS1 (of the other subunit) and then from cFeS2 (also of the other subunit) to FMN (of the same subunit as FAD and nFeS1 and nFeS2). The redox potentials of the four [4Fe-4S] clusters of DPD have not been determined nor were those of the flavin coenzymes. However, only two of the [4Fe-4S] clusters could only be reduced photochemically along with the two flavins (estimated *E*_m_, −0.44 V, pH 9.5 [111,112]) and were identified with those in the C-terminal 8Fe Fd module. Furthermore, only small amounts of Sq forms were detected during reductive titrations, suggesting that, as in GltS, the midpoint potential of at least one of the flavin Sq/Hq couples is at least 120 mV higher than that of the corresponding Ox/Sq pair.

Thus, in the absence of redox potential changes of the cofactors during the catalytic cycle, thermodynamically uphill electron transfer steps must take place during the DPD reaction when the two electrons loaded on FAD as a hydride anion from NADPH are transferred to the chain formed by the four [4Fe-4S] clusters. The complexity of DPD may be the result of assembling pre-existing domains (a GltD-like NADPH oxidizing FAD-containing enzyme and a DHODH-like module) and ensuring electronic communication using two additional [4Fe-4S] clusters. However, it may also have evolved in order to finely tune the reaction. Low potential Fe-S clusters may avoid over-reduction of the enzyme, which just needs to be able to transfer two electrons as a hydride anion from NADPH to uracil. Indeed, NADPH titrations of DPD led to absorbance changes consistent with only 0.5 flavin being reduced, while the presence of an NADPH-regenerating system formed by glucose 6-phosphate dehydrogenase and glucose 6-phosphate led to the reduction of both flavins. Only photoreduction brought along also the reduction of two of the four [4Fe-4S] clusters. The low potential of the two [4Fe-4S] clusters of the GltD-like domain could be only partially explained by structure determination, which revealed the presence of a glutaminyl ligand of nFeS2. The latter is a glutamate in GltS [4Fe-4S]-I cluster (figure 10) [96,97].

The Fe-S clusters of DPD may also help to guarantee the cross-talk between the catalytic subsites at a distance. Interestingly, the crystal structure of DPD that had been incubated with NADPH showed a repositioning of the loop of the uracil reducing (FMN) domain such that the catalytically essential C671 becomes suitably positioned to promote uracil reduction by donating a proton to the C5 position [109]. On the other hand, using the catalytically inactive C671A-DPD variant, which can bind the pyrimidine substrate, but is unable to complete its reduction, it was shown that it is only in the presence of the uracil substrate that NADPH oxidation takes place at a rate sufficiently high to support turnover [112].

**Figure 13.**
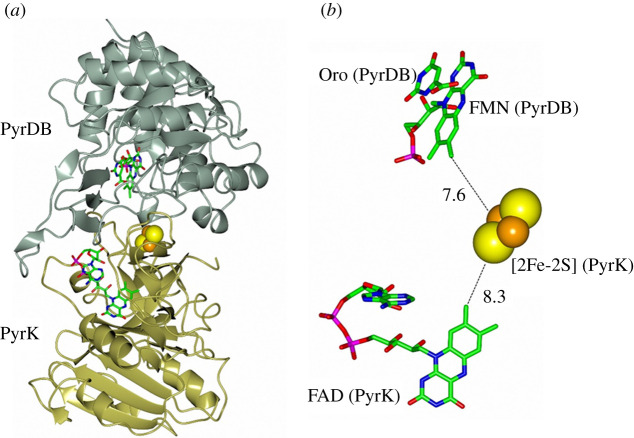
*Lactococcus lactis* DHODH-1B in complex with the orotate substrate. (*a*) Ribbon representation of the PyrK (gold)-PyrDB (grey-green) protomer of *Lactococcus lactis* DHODH-1B (PDB ID: 1EP2 [114]) with FAD and FMN coenzymes in sticks and the [2Fe-2S] cluster atoms in spheres. (*b*) Details and distances (in Å) of the redox centres and the orotate (ORO) ligand.

Interestingly, the FMN-containing domain, where uracil binds and is reduced, is structurally and mechanistically related to DHODH, which catalyse the oxidation of dihydroorotate to orotate in a reaction that may be seen as the reverse of the DPD one. There are actually two main classes of DHODH [113]. Class 2 enzymes are single-subunit, membrane-bound, proteins, and complete the catalytic cycle by reducing CoQ fuelling the respiratory chain. Class 1 enzymes are divided into two subclasses 1A and 1B. DHODH-1A is a homodimer and is reoxidized by fumarate. DHODH-1B is instead a two subunit protein formed by an FMN-containing *α*_8_*β*_8_ barrel PyrDB subunit similar to DHODH-A (and DPD uracil reducing (FMN) domain), and a second PyrK subunit containing one [2Fe-2S] cluster and FAD, which mediates the transfer of electrons extracted from dihydroorotate to NAD^+^ (figure 13) [114]. The latter is unrelated to GltS *β* subunit and the N-terminal domain of DPD. Rather, it is reminiscent of PDR architecture with a FNR module followed by a C-terminal domain carrying the [2Fe-2S] cluster [92]. However, such C-terminal domain is unrelated to that of PDR. Interestingly, as in GltS, also in DHODH-1B, the PyrDB subunit appears to be required for the incorporation of the FeS cluster in PyrK. However, in this case, also binding of FAD to the FNR-like module of PyrK seems weakened in the absence of PyrDB, and also the PyrDB subunit is destabilized in the absence of PyrK [115,116].

**Figure 14.**
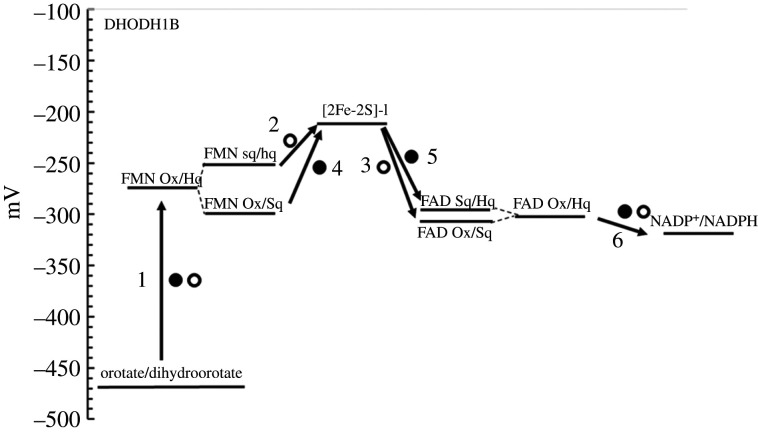
Proposed electron pathway for *Lactococcus lactis* DHODH-1B. The first and second electron leaving the FMN coenzyme are shown as empty and full circles, respectively. The numbers are meant to guide the reader through the sequence of electron transfer steps.

Also in DHODH-1B thermodynamically favoured (downhill) and unfavoured (uphill) steps may alternate within an overall favoured redox reaction (*E*_m_ for the orotate/dihydroorotate couple, *ca* −470 mV and for NADP^+^/NADPH −320 mV). Both FMN and FAD Ox/Sq and Sq/Hq couples are separated by only approximately 40 mV allowing for the accumulation of a small amount of neutral Sq for both FMN and FAD (*E*_1_, −301 mV for the addition of the first electron to the FMN coenzyme producing FMN Sq, and *E*_2_, −252 mV for the addition of the second electron producing FMN Hq; *E*_1_, −312 mV and *E*_2_, −297 ± 5 mV for FAD Ox/Sq and Sq/Hq couples, respectively). With a midpoint potential of −212 mV for the [2Fe-2S] cluster, the electron transfer from FMN to the Fe-S cluster is favoured, but that from the Fe-S to FAD is not. Also in this case, the bound substrate/product couples have been proposed to modulate the redox potentials of each centre during the catalytic cycle (figure 14) [115,117].

### 6.3. Nfn and the flavin-based electron bifurcation mechanism

FBEB is a recently discovered mechanism of biological energy conservation in enzymes of strictly anaerobic bacteria and Archaea, which conceptually parallels the logics of the quinone-based electron bifurcation of the well-characterized Q cycle. The two electrons donated one at a time by the reduced quinone to the Rieske-type Fe-S cluster of complex III follow different routes: one electron is passed to cytochrome c_3_ and then to cytochrome c, while the other is transferred from the Sq back to another quinone molecule through haem b_L_ and haem b_H_. In FBEB, a flavin coenzyme is located at the electron entry site and accepts two electrons from NAD(P)H, F420, formate or molecular hydrogen. The flavin Hq is reoxidized in two 1-electron transfer steps, as it is often observed in the examples of Fe-S flavoenzymes mentioned so far. However, the two electrons follow different paths as in the case of the Q cycle in Complex III or as initially proposed for GltS [41]. The first electron leaving the flavin Hq can reduce a high potential acceptor in a thermodynamically favoured process. The electron transfer from the bifurcating flavin to the high potential acceptor takes place with the intermediacy of one or more Fe-S clusters and a flavin at the end of the chain. It is at the end of two catalytic cycles that such flavin is in the Hq state and can reduce the high potential electron acceptor, which can be NAD(P)^+^, crotonylCoA, caffeylCoA, pyruvate, the CoM-SS-CoB heterodisulfide or a quinone. The second electron ‘left behind’ on the flavin Sq—at a low potential—follows a different route and reduces a low potential electron acceptor, typically a Fd, through another intramolecular electron transfer chain formed by one or more Fe-S clusters. Several of the reactions are reversible giving rise to a confurcation mechanism so that, in this back reaction, two 1-electron donors make the electrons converge on the bifurcating flavin coenzyme.

The reader is referred to several recent excellent reviews for overviews and discussions of the biological meaning of FBEB, and the properties of the bifurcating enzymes also in a historical and evolutionary perspective [19,21,22,118–122].

Briefly, the well-characterized enzymes that adopt the FBEB mechanism consist of four unrelated families that contain (i) electron-transferring flavoproteins (EtfAB), (ii) NAD(P)H dehydrogenase (NuoF homologues), (iii) heterodisulfide reductase (HdrABC) or HdrABC homologues and (iv) NADH-dependent Fd : NADP reductase (NfnI or NfnAB). Among such enzymes, the latter appears to be the simplest, and the *P. furiosus* (NfnI) and *T. maritima* (NfnAB) forms are the best-characterized ones [123–126]. Interestingly, *P. furiosus* NfnI is identical to sulfide dehydrogenase, which has been previously characterized by different groups [94,95,127].

**Figure 15.**
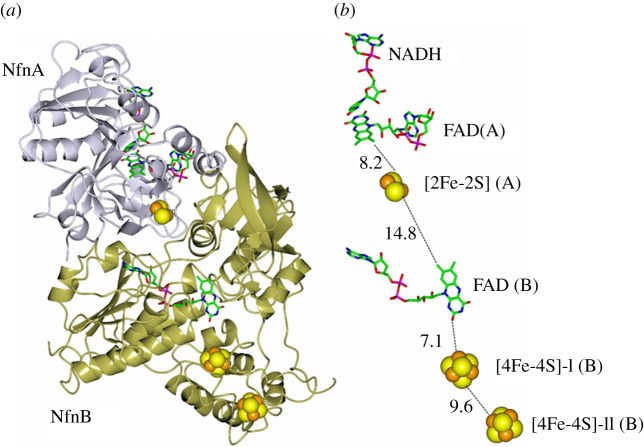
*Thermotoga maritima* NfnAB structure and the FBEB. (*a*) Ribbon model of the NfnB (gold)-NfnA (grey) protomer of *T. maritima* NfnAB in complex with NADH (PDB ID: 4YRY [124]) with the Fe-S clusters as spheres, the FAD coenzymes and bound NADH in sticks. (*b*) Details of the redox centres and the NADH ligand with the indication of the subunit to which they are bound and distances in Å.

For simplicity, the *T. maritima* nomenclature will be adopted. NfnAB is formed by one small subunit (NfnA, corresponding to *P. furiosus* NfnI-S, approx. 32 kDa), which harbours one [2Fe-2S] cluster and one FAD, and a large NfnB subunit (corresponding to *P. furiosus* NfnI-L, approx. 50 kDa). The latter, corresponding to sulfide dehydrogenase SudA subunit, belongs to the class of GltD-like NAD(P)H oxidoreductases containing two low potential [4Fe-4S] clusters and one FAD. The NfnA subunit (previously identified as the sulfide dehydrogenase SudB subunit) is, instead, structurally related to DHODH-1B PyrK subunit with a FAD-binding region belonging to the Fd : NADP reductase family of flavoenzymes and the C-terminal module containing the [2Fe-2S] cluster (figures 13 and 15). In the reaction, FAD of the GltD-like NfnB subunit (FAD-b) is the ‘bifurcating flavin’ and is reduced by NADPH. A first electron at the high potential of the Sq/Hq couple is transferred from FAD-b on the B subunit to the FAD coenzyme at the NAD^+^ reducing site on NfnA (FAD-a) via the [2Fe-2S] cluster also on the A subunit. The second electron, thanks to the low potential of the Ox/Sq couple of FAD-b on NfnB, can be transferred via the two GltD-like low potential [4Fe-4S] clusters to Fd [124]. It is at the end of two cycles that one molecule of NADH is formed. Such structure-based mechanism was initially supported by taking into account the redox potential values on which the bifurcated electron transfer pathway was proposed for GltS [41,124] (figure 11) for the NfnB redox centres, namely, for FAD-b values of the Ox/Sq couple of less than or equal to −360 mV and of the Sq/Hq couple greater than or equal to −240 mV (corresponding to a potential value of approximately −300 mV for the Ox/Hq couple), and the [4Fe-4S] clusters tentatively assigned potentials of approximately −400 mV.

The redox potential of the [2Fe-2S] cluster of the *NfnA/* NfnIS subunit [previously known as SudB] was experimentally determined and found to be unusually high (+80 mV) with an also unusual Asp(Cys)_3_ ligation pattern [124] confirming previous findings [127]. Structure determination also demonstrated the presence of the two GltD-like [4Fe-4S] clusters, one of which has a Glu (Cys)_3_ ligation pattern as in GltS, correcting the initial identification of one [4Fe-4S] and one [3Fe-4S] cluster in SudA. Initial estimates of the redox potential of FAD bound to the NfnA subunit at the NAD^+^ reducing site were obtained by taking into account the values experimentally determined for DHOHD-B PyrK subunit (for FAD: Ox/Sq, −312 mV, Sq/Hq, −297 mV; Ox/Hq, −304 mV figure 14). With these assumptions, the redox potential values would be consistent with an overall electron transfer from NADPH to NAD^+^, especially by taking into account estimates of the redox potentials of the pyridine nucleotides under physiological conditions (for NADP^+^/NADPH, −370 mV, for NAD^+^/NADH, −280 mV rather than −320 mV for both), and to Fd (−400 mV).

Experimentally determined distances between the redox centres are also consistent with fast electron transfer, even in the predicted thermodynamically uphill steps. An exception is the 15 Å distance that separates the bifurcating FAD and the [2Fe-2S] centre, which is considered ‘borderline’. However, small rearrangements in the intersubunit contacts, as a consequence of NADPH binding and bifurcating flavin reduction, have been proposed to transiently lower it. Thus, protein dynamics, besides the potential values, may control the direction and rate of electron transfer in the exoergonic and endoergonic branches of the process. The latter hypothesis was fully supported by hydrogen/deuterium exchange mass spectrometry experiments [123,125].

**Figure 16.**
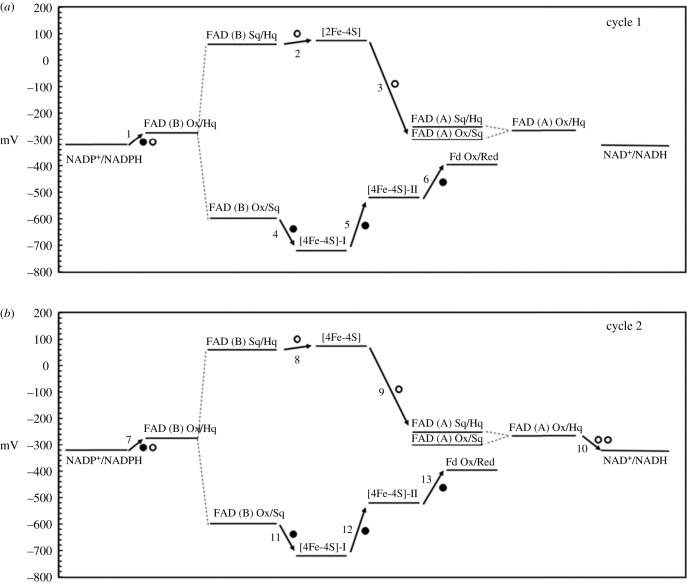
Proposed electron transfer pathway in NADH-dependent reduced ferredoxin: NADP^+^ oxidoreductase (Nfn). From the measured and calculated potential values of the redox centres of *P. furiosus* NfnI and *T. maritima* NfnAB, the reduction of NAD^+^ in the NfnA (or NfnIS) subunit requires two cycles of NADPH oxidation at the NADPH oxidizing site containing the bifurcating flavin in the GltD-like NfnB (or NfnIL) subunit with concomitant reduction of two Fd molecules [124,126]. Note that only a 600 mV separation in the potential for the bifurcating FAD Ox/Sq and Sq/Hq couples is indicated instead of the initially proposed 1.2 V value [126], as discussed in [21]. With this assumption, the number of thermodynamically uphill steps is minimized. The numbers help following the path of the first and second electron leaving the bifurcating flavin shown as empty and full circles, respectively.

More recently, estimates of the redox potentials of *P. furiosus* NfnI redox centres were obtained [126] (figure 16). The *E*_m_ value of the Ox/Hq couple of both the bifurcating FAD (on NfnI-L, the *T. maritima* NfnB) and that at the NAD^+^ reducing site (on NfnI-S or NfnA) was estimated to be −276 mV. NADH was found to be able to reduce the FAD at the NAD^+^ reducing site on the NfnI-S subunit, and the [2Fe-2S] cluster leading to a stable FAD-L neutral Sq, which appears to electronically interact with the reduced [2Fe-2S] cluster as found for DHODH-B. From the Sq stability, the redox potentials of FAD on the NfnI-L/NfnA subunit Ox/Sq and Sq/Hq couples were estimated to be −301 mV and −252 mV, respectively (potential difference, only 50 mV), well in the range required for overall reduction by NADPH and for reducing NAD^+^.

Square wave voltammetry measurements yielded estimates of the midpoint potential of −513 mV and −718 mV for the two [4Fe-4S] clusters at pH 8. The lower value of −718 mV was assigned to the [4Fe-4S] cluster proximal to the bifurcating flavin, and the other (−513 mV) to the distal one, generating a thermodynamically favoured electron transfer path to Fd (*P. furiosus* Fd potential, approx. −400 mV). Transient UV-visible absorption spec­troscopy was used to estimate the values of the potential of the Ox/Sq and Sq/Hq couples for the bifurcating FAD on NfnI-L (or NfnB). Irradiation of the NADPH-reduced NfnI revealed the appearance of a short-lived anionic Sq that was assigned to (transient) electron transfer from the 2-electron reduced bifurcating flavin and the proximal [4Fe-4S] cluster. By taking into account the lifetime of the Sq, the calculated potential of the [4Fe-4S] cluster (−718 mV) and the geometry of the protein, as derived from the crystal structure, it was proposed that the Ox/Sq and Sq/Hq couples of the bifurcating flavin are separated by 1.2 V. Thus, for the bifurcating FAD, the estimated midpoint potential values were −911 mV for the Ox/Sq couple and +359 mV for the Sq/Hq couple at pH 8. However, by taking into account the experimental setting, it has been suggested that the potential of the Ox/Sq couple may actually be less negative by approximately 300 mV, leading to values of the Ox/Sq couple in the range of −600 mV and of the Sq/Hq couple in the +59 mV range with a separation of only 600 mV, a value that is still remarkable and consistent with the proposed mechanism alternating thermodynamically downhill and uphill steps in both exoergonic and endoergonic branches of the reaction [21]. Furthermore, it has been argued that the consequences of the conformational changes upon substrate binding that have been detected by a series of hydrogen/deuterium mass spectrometry-based experiments [123,125] on the properties of the redox centres, and their distances, need to be determined to clarify thermodynamics and kinetics of the electron transfer path in NfnI enzymes [21].

Interestingly, the second NADH-dependent Fd : NADP^+^ reductase of *P. furiosus* (NfnII) is also a Fe-S flavoenzyme formed by a large (NfnII-L) and a small (NfnII-S) subunit, similar to NfnI/NfnAB large (B) and small (A) subunits, respectively. Surprisingly, activity assays and structure determination showed that it does not appear to be an ‘electron bifurcating’ enzyme endowed of a NADPH/NAD^+^ transhydrogenase activity coupled to the Fd reductase one. Rather, it exhibits a ‘simple’ NADPH : Fd reductase activity due to the fact that the NADH binding site on the NfnA (Nfn-IIS) subunit is obstructed by a loop, corresponding to insertion in NfnII-L primary structure as compared to NfnI. Moreover, several residues that are important for NAD^+^ binding have been changed. Whether a high potential acceptor different from NAD^+^ is used in an FBEB mechanism also in this enzyme is not known yet [95].

### 6.4. Bacterial AegA, a GltD-like containing protein of unknown function

*E. coli* AegA is the last and least understood member of proteins adopting the GltD-like module [93]. From primary structure analyses, in AegA and in the closely related YgfT, the GltD-like domain is preceded by an N-terminal domain similar to bacterial [4Fe-4S] Fds. The presence of the GltD-like domain and the similarity of such Fd module with components of hydrogenases and formate dehydrogenases guided experiments that recently led to propose that AegA participates in formate- and formate dehydrogenase H-dependent reductive uric acid degradation [128]. By analogy with GltS and DPD, AegA may reduce an acceptor in the uric acid degradation pathway in a NAD(P)H-dependent reaction or, by analogy with NfnAB, AegA may belong to the flavin-based bifurcating enzymes class and use reduced Fd from formate dehydrogenase to carry out its reaction(s). Thus, the understanding of AegA function and mechanism awaits further experimental work.

## 7. Conclusion and future perspectives

The present overview of a limited, and rather arbitrary, selection of Fe-S flavoenzymes shows, once more, the versatility of Fe-S clusters and flavin coenzymes so that by mixing and matching protein modules a great variety of functions is obtained. However, it is also evident how our understanding of the structure−function relations of enzymes is still relatively limited because of the large effects of fine details of protein structure on the actual function and mechanism of action of each protein. Therefore, generalizations based on sequence similarities, and even high-resolution structures, can only provide a starting point for sound experimental work aimed to determine the actual mechanism of action of the protein of interest, as assisted by the variety of other *in silico* and *in vivo*/*ex vivo* approaches that are now available, and often preferred over more traditional biochemical and biophysical work. The high rates of electron transfer along with the need of sophisticated spectroscopic methods make it difficult to complement equilibrium information with key data on the actual rates of individual reaction steps.

Besides the experimental identification of the actual function and the determination of the properties and location of the redox centres, a challenging aspect in all these enzymes remains the experimental determination of how, even limited, protein conformational changes following subunit–subunit interactions and, especially, during the catalytic cycle affect electron transfer. The comparison of experimentally determined rates with those predicted by theory from structural models and thermodynamics may indeed allow to detect the presence of conformational changes that affect electron transfer. Progress in high-resolution time-resolved structural studies may eventually help to overcome some of these issues in an increasing number of cases, eventually leading to direct observation of enzymes in action, including the broad complex and growing class of Fe-S flavoenzymes.

## Abbreviations

2-IG, 2-iminoglutarate; 2-OG, 2-oxoglutarate; α-GltS, glutamate synthase *α* subunit; β-GltS, glutamate synthase *β* subunit; APS, adenosine 5′-phosphosulfate; CoQ, coenzyme Q; DHODH, dihydroorotate dehydrogenase; FBEB, Flavin-based electron bifurcation; DPD, dihydropyridine dehydrogenase; GltS, glutamate synthase; EXAFS, Extended X-ray absorption fine structure; *E*_m_, midpoint potential; FAD, flavin adenine dinucleotide; FMN, flavin mononucleotide; FNR, Fd : NADP^+^ oxidoreductase; Fd, Fd; Fd-GltS, Fd-dependent glutamate synthase; Fe-S, iron-sulfur; Ox, oxidized; Sq, semiquinone; Hq, hydroquinone; Nfn, NADH-dependent reduced Fd : NADP^+^ oxidoreductase; NADPH-GltS, NADPH-dependent glutamate synthase; PDR, phthalate dioxygenase reductase; Rf, riboflavin; RK, riboflavin kinase; FADS, FAD synthetase; Fld, flavodoxin, FumR, fumarate reductase; ETF, electron-transferring flavoprotein; EPR, electron paramagnetic resonance; XOR, xanthine oxidoreductase; XO, xanthine oxidase; XDH, xanthine dehydrogenase; SDH, succinate dehydrogenase; TMADH, trimethylamine dehydrogenase.

## Data accessibility

This article does not contain any additional data.
